# Hyaluronan fragments induce IFNβ via a novel TLR4-TRIF-TBK1-IRF3-dependent pathway

**DOI:** 10.1186/1476-9255-10-23

**Published:** 2013-05-30

**Authors:** Katharine E Black, Samuel L Collins, Robert S Hagan, Mark J Hamblin, Yee Chan-Li, Robert W Hallowell, Jonathan D Powell, Maureen R Horton

**Affiliations:** 1 Department of Medicine, John Hopkins University School of Medicine, Baltimore, USA; 2 Department of Oncology, Johns Hopkins University School of Medicine, Baltimore, USA

**Keywords:** Hyaluronan, Macrophage, Interferon, Matrix, Lung

## Abstract

**Background:**

The extracellular matrix plays a critical role in insuring tissue integrity and water homeostasis. However, breakdown products of the extracellular matrix have emerged as endogenous danger signals, designed to rapidly activate the immune system against a potential pathogen breach. Type I interferons play a critical role in the immune response against viral infections. In the lungs, hylauronan (HA) exists as a high molecular weight, biologically inert extracellular matrix component that is critical for maintaining lung function. When lung tissue is injured, HA is broken down into lower molecular weight fragments that alert the immune system to the breach in tissue integrity by activating innate immune responses. HA fragments are known to induce inflammatory gene expression via TLR-MyD88-dependent pathways.

**Methods:**

Primary peritoneal macrophages from C57BL/6 wild type, TLR4 null, TLR3 null, MyD88 null, and TRIF null mice as well as alveolar and peritoneal macrophage cell lines were stimulated with HA fragments and cytokine production was assessed by rt-PCR and ELISA. Western blot analysis for IRF3 was preformed on cell lysates from macrophages stimulate with HA fragments

**Results:**

We demonstrate for the first time that IFNβ is induced in murine macrophages by HA fragments. We also show that HA fragments induce IFNβ using a novel pathway independent of MyD88 but dependent on TLR4 via TRIF and IRF-3.

**Conclusions:**

Overall our findings reveal a novel signaling pathway by which hyaluronan can modulate inflammation and demonstrate the ability of hyaluronan fragments to induce the expression of type I interferons in response to tissue injury even in the absence of viral infection. This is independent of the pathway of the TLR2-MyD88 used by these matrix fragments to induce inflammatory chemokines. Thus, LMW HA may be modifying the inflammatory milieu simultaneously via several pathways.

## Background

The type I interferons have been implicated in a wide variety of host defense responses [1]. Though there are many type I interferons in humans (α, β, ω, κ, ϵ), IFNβ and IFNα are the best described [1]. Multiple cell types produce each interferon, making the original distinction between “leukocyte” IFN (alpha) and fibroblast IFN (beta) obsolete [1]. These type I interferons share a common receptor, the interferon-α/β receptor [1]. IFNs induce antiviral cellular responses to diverse stimuli, including LPS, *Shigella*, *Plasmodium*, *Schistomsoma*, *Mycoplasma*, trypanosomes, as well as viruses such as RSV [2-4].

Although cells usually produce IFNα and β in response to viral or other infections, the interferons play a role in the absence of infection as well [5,6]. IFNβ production by plasmacytoid dendritic cells and Th1 T cells has been implicated in inhibiting Th2 cell migration in inflamed lungs in a model of allergic asthma [5]. Furthermore, low level IFN production has been observed in the absence of infection both *in vivo* and *in vitro*[6]. It has been proposed that this low level IFN production is necessary for cells to mount a rapid and effective response to infections by priming the cells for further, rapid IFN responses [6].

Hyaluronan (HA) is a glycosaminoglycan found throughout the extracellular matrix. It plays a variety of roles both in maintaining structure and in responding to injury [7-9]. HA is produced predominantly in fibroblasts by three isoforms of hyaluronan synthase, and is released from the plasma membrane into the extracellular space [7]. It is abundant in the synovial and vitreous fluids and makes up 80% of the glycosaminoglycan in the lung [7]. In a healthy lung, HA exists predominantly in a high molecular weight form (6 million daltons) that is important in maintaining distribution of plasma proteins. High molecular weight HA is immunosuppressive by a variety of mechanisms including enhancing suppressive T regulatory cells, and inhibiting macrophage phagocytosis [7,10]. However, fragments of HA produced both by breakdown of high molecular weight forms and by direct synthesis by hyaluronan synthases have profound biological activities that diverge from these pro-homeostatic effects [8,9,11]. In the setting of tissue destruction, low molecular weight fragments of hyaluronan (200,000 daltons) accumulate and act as an endogenous “danger signal”, inducing a host of inflammatory mediators [12-15]. These fragments have been shown to act via TLR2-MyD88-IRAK–PKζ or TLR4-MyD88 binding to promote the production of inflammatory chemokines, such as MIP-1α, MIP 1β, KC, RANTES, MCP-1, IP-1, IL-12, IL-8 and TNFα [12-15].

In light of the emerging importance of hyaluronan in regulating inflammation in response to tissue injury, we sought to determine its potential role in regulating the induction of type I interferons. In this report we demonstrate the ability of HA fragments to promote IFNα and β expression in the absence of viral infection. Furthermore, our studies reveal a previously unappreciated MyD88-independent signaling pathway responsible for HA-induced inflammatory gene expression. Thus, LMW HA may be modifying the inflammatory milieu simultaneously via several pathways.

## Methods

### Cells

MH-S cells (a murine alveolar macrophage line) or RAW 264.7 cells (a murine leukemic monocyte/macrophage line derived from ascites) were used (ATCC) [16]. Cells were cultured in RPMI or DMEM media with 10% fetal bovine serum. For experiments, cells were washed in phosphate-buffered saline and stimulated in RPMI 1640 media with 1% glutamine and penicillin/streptomycin. Thioglycollate-elicted peritoneal macrophages were lavaged from female C57BL/6, TLR2 null, TLR4 null, TRIF null, TLR3 null (The Jackson Laboratory), or MyD88 null (Akira and Gollenbeck) mice 4 days after injection of 3 mL sterile thioglycollate (Sigma-Aldrich). Before use, cells were allowed to adhere overnight in RPMI 1640 supplemented with 10% heat inactivated low-LPS FBS and 1% penicillin-streptomycin/glutamine. To exclude the effects of contaminating LPS on experimental conditions, cell stimulation was conducted in serum-free RPMI, and in the presence of polymixin B at 10 μg/mL (Sigma-Aldrich) unless LPS was used as a stimulant. All animal experiments were approved by the Johns Hopkins Committee on Animal Use and experiments were conducted in accordance with their guidelines and regulations.

### Chemicals and reagents

Purified LMW HA fragments (free of protein and other glycosaminoglycans), with a peak molecular weight of 200,000 Da derived from human umbilical cords, were purchased from Calbiochem. Polymixin B was purchased from Sigma. Ultrapure LPS was purchased from InvivoGen. HMW HA was purchased from Genzyme. HA disaccharides and chondroitin sulfate B were purchased from Sigma. BX795 was purchased from Axon, Medcom.

### RT-PCR

Total RNA was collected in Trizol reagent (Invitrogen), stored at -20°C. RNA was extracted per manufacturer’s protocol. Purity and RNA concentration was measured using a NanoDrop (ThermoScientific). cDNA was prepared using Superscript III (Invitrogen) according to manufacturer’s protocol. Real Time PCR was done using an ABI 7900 cycler and commercially available composite probesets for interferon beta (ABI). Eukaryotic 18S was used as internal control. Data were presented as fold induction over unstimulated samples; for knockout mice experiments, data are presented as fold induction over wild-type unstimulated samples.

### Luciferase

The human IFN-beta promoter luciferase reporter plasmid PGL-3-IFN-beta-LUC41 was a kind gift of J. Hiscott (McGill University, Montreal, Canada). RAW 264.7 cells were transfected with 0.5 ug of plasmid per 3million cells using lipofectamine 2000 (Invitrogen); cells were transfected for 4 hours, then allowed to rest in complete media overnight and replated in 96 or 12 well plates. Cells were then rinsed in warmed PBS and stimulated in serum-free RPMI with polymixin B (Sigma). Luciferase activity was measured using Bright-Glo (Sigma) and a microplate reader.

### ELISAs

Cell cultures were stimulated with HA for the allotted time; supernatants were collected and stored at -80°C. until the ELISAs were performed. ELISAs for interferonβ (PBL) were performed according to manufacturer’s specifications.

### Western blot analysis

10 ug of whole cell lysates were fractionated by SDS-PAGE (10%), transferred to nitrocellulose membrane, blocked with 5% milk, washed, and incubated with primary antibodies to IRF-3 (1:1000) or phospho-IRF3 S396(1:1000) (Santa Cruz Biotechnology). Secondary antibodies were purchased from GE Healthcare and developed with a chemiluminescent system according to the manufacturer’s instructions (Amersham).

### Statistics

All statistical analysis was conducted using the Student’s t-test. Statistically significant values were considered to be those where p < 0.05.

## Results

### Low molecular weight HA fragments induces IFNβ

**Figure 1:**
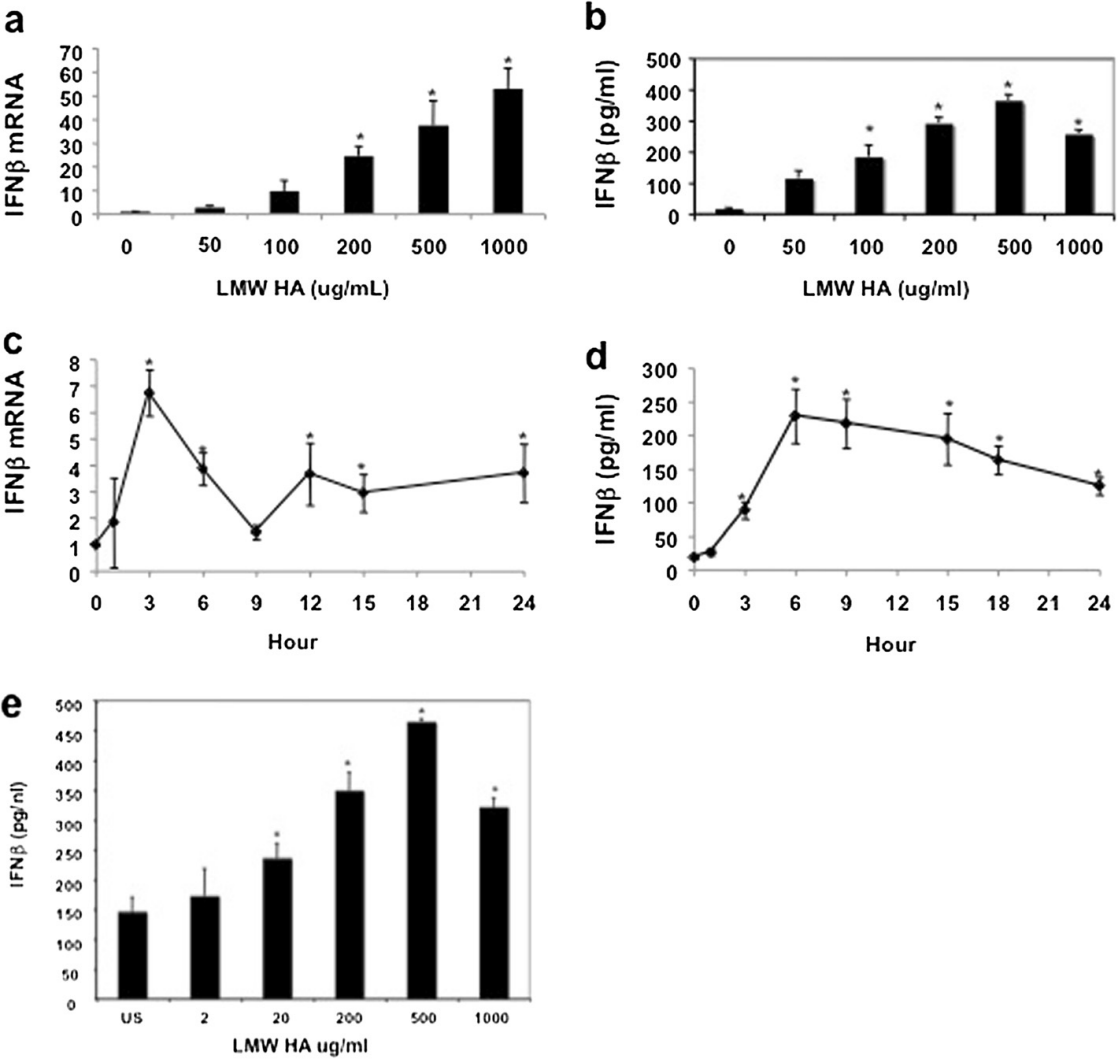
**HA fragments induce IFNβ mRNA and protein.** (**a**) MH-S alveolar macrophages were stimulated with low molecular weight (LMW) HA fragments, RNA extracted and Real-Time PCR performed for IFNβ normalized to 18 s. HA fragments induce IFNβ mRNA in a dose dependent fashion with peak induction at 3 h with a dose 1000 μg/ml. (**b**) HA fragments induce IFNβ protein in RAW 264.7 macrophages in a dose dependent fashion with peak induction at 6 h with a dose of 500 μg/ml. (**c**,**d**) HA fragments induce IFNβ RNA and protein in peritoneal macrophage cell line in a time dependent fashion with peak induction mRNA at 3 h and protein at 6 h in macrophages; cells were stimulated with 200 μg/ml of HA fragments. (**e**) Thioglycollate elicited primary mouse macrophages from C57BL/6 WT mice were stimulated with HA fragments (200 μg/ml) for 6 h and IFNβ protein secretion was measured by ELISA. These figures are representative of at least 3-4 identical experiments done in triplicate. ^★^ p ≤ 0.05 vs. unstimulated.

In light of the critical role of Type I IFN in regulating infectious and non-infectious immune responses we sought to determine if endogenous HA fragments could act as a danger signal by inducing IFNβ in macrophages. Both alveolar macrophage and peritoneal macrophage cell lines were stimulated with HA fragments in serum-free RPMI. Total RNA and protein were isolated, and analyzed with quantitative PCR or ELISA. HA fragments induced IFNβ mRNA and protein in a dose-dependent fashion, with peak protein expression after 500 ug/ml HA (Figure 1a,b). IFNβ mRNA demonstrated a peak increase at 3 hour and peak protein at 6 h (Figure 1c,d). Of note, this peak occurred before HA fragment induced production of other inflammatory cytokines such as TNF-α, suggesting that the production of IFNβ is a primary effect of HA, not a downstream effect of TNF-α stimulation [17]. Additionally, the ability of HA fragments to induce IFNβ was observed in primary peritoneal macrophages indicating that our results were not confined to cultured cell lines (Figure 1e). Thus, HA fragments can induce the induction of IFNβ RNA and protein in both macrophage cell lines and primary macrophages.

### Specific induction of IFNβ by HA fragments

**Figure 2:**
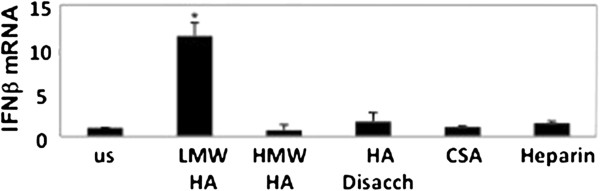
**HA fragments induce IFNβ in a specific fashion.** RAW 264.7 macrophages were stimulated with LMW HA fragments (200 μg/ml) for 3 h, RNA extracted and Real-Time PCR performed for IFNβ. HA fragments but not HMW HA, HA disaccharides, chondroitan sulfate A (CSA) or heparin induce IFNβ mRNA. This figure is representative of at least 3 identical experiments done in triplicate. ^★^ p ≤ 0.01 vs. unstimulated.

In the lung at rest, HA exists in a high molecular weight form that plays roles in maintaining tissue integrity and water homeostasis [7]. We have previously shown that upon tissue damage the HA is broken down into lower molecular weight fragments, and that only this form acts as an endogenous danger signal by activating the innate immune response [9]. To determine if hyaluronan induction of IFNβ is specific to HA fragments, macrophages were stimulated with HA fragments, high molecular weight HA, or other glycoasminoglycans in serum free RPMI for 3 hours. Total RNA was then isolated and analyzed by quantitative PCR. As predicted, HA fragments but not high molecular weight HA induced the production of IFNβ Figure 2). Furthermore, other glycosaminoglycans such as HA disaccharides, chrondroitan sulfate A (CSA), and heparin, failed to induce IFNβ mRNA expression (Figure 2).

### TLR4 but not TLR2 is required for HA fragment induction of IFNβ

**Figure 3:**
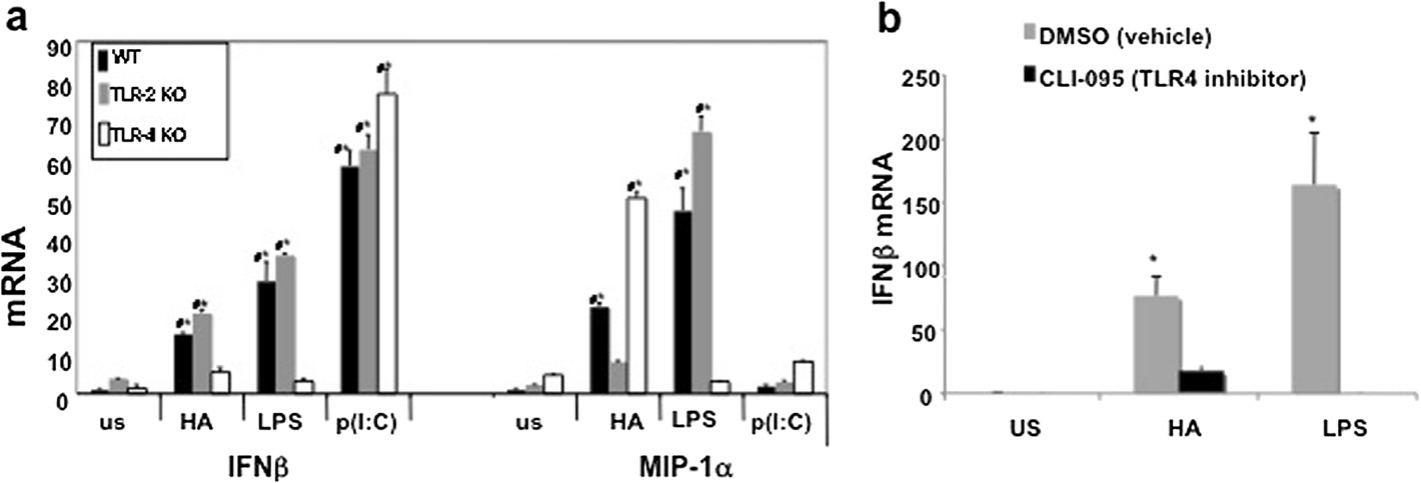
**HA fragment induction of IFNβ is TLR-4 dependent.** (**a**) Thioglycollate elicited peritoneal macrophages from C57BL/6 WT, TLR2 receptor null or TLR4 receptor null mice were stimulated with 200 ug/mL LMW HA fragments for 3 hours; cells were collected in Trizol, RNA extracted, and cDNA was analyzed with real-time PCR. HA fragment-induced MIP1α requires TLR2; HA induction of IFNβ was independent of TLR2 but required TLR4. Data demonstrate IFNβ induction compared to WT unstimulated levels; ^★^ p < 0.05 compared to WT unstimulated; # = p <0.05 compared to genotype specific unstimulated). (**b**) Pharmacological inhibition of TLR4 significantly decreases HA fragment activation of IFNβ. RAW 264.7 macrophages were prestimulated with 1 ug/mL CLI-095 for thirty minutes, prior to HA fragments (200 ug/ml) or LPS (100 ng/ml) for 3 hours. Cells were collected in Trizol, RNA extracted, and cDNA was analyzed with real-time PCR. Blocking the TLR4 receptor with CLI-095 significantly inhibited both HA fragment and LPS induction of IFNβ. Values shown are mean fold induction over WT unstimulated; These figures are representative of at least 3-4 identical experiments done in triplicate ^★^ p < 0.05 unstimulated stimulated.

We have previously demonstrated that HA fragments induce inflammatory cytokines via a TLR2-MyD88-dependent pathway [9]. Others have implicated TLR4, or the combination of TLR2 and TLR4 in response to hyaluronan fragments of various sizes [8,18]. TLR4, TLR3, and TLR9 are well established as inducers of IFNβ expression. Although recently vaccinia has been reported to induce IFNβ via TLR2, in general TLR2 signaling pathways do not induce IFNβ. [19-24]. In some models TLR2 agonists actually inhibit IFNβ production [25]. Thus, we wanted to determine which, if any, of the TLR receptors was involved in mediating HA-induced IFNβ expression. Thiolglycollate-elicited primary macrophages were harvested from TLR2 null, TLR4 null, and wild type (WT) mice and stimulated in serum-free media with 200 ug/mL of LMW HA for 3 hours. RNA was extracted and analyzed by real time quantitative PCR. Both TLR2 null and WT macrophages responded briskly to HA fragments, with a marked and significant increase in IFNβ mRNA. However, the TLR4 null macrophages failed to produce IFNβ after LMW HA stimulation (Figure 3a). In contrast, LMW HA-induced MIP1α production was reduced in macrophages from TLR2 null mice but robustly induced by macrophages lacking TLR4. In addition, macrophages from both TLR2 and TLR4 null mice demonstrated robust expression of IFNβ in response to poly (I:C), a TLR3 agonist.

TLR2 signaling thus proved to be necessary for the chemokine expression, but not the IFNβ expression induced by HA fragments. In contrast, TLR4 appeared responsible for HA-induced IFNβ expression but not chemokine expression. Thus, the ability of HA fragments to stimulate the C-C chemokine MIP1α and IFNβ is by two distinct pathways: HA fragments induced chemokine expression via TLR2 while the same HA fragments induced type I interferon via TLR4.

To confirm the dependence of HA fragment-induced IFNβ on TLR4 we employed the pharmacologic inhibitor CLI-095 [26]. This molecule blocks TLR4 activation without inhibiting other MyD88-dependent signaling. Macrophages were pre-incubated with either CLI-095 or DMSO as a control for 30 minutes, stimulated with HA fragments or LPS for 3 hours, and then evaluated for IFNβ expression by RT PCR. Consistent with the observations in the TLR4 null macrophages, CLI-095 inhibited HA fragment induction of IFNβ mRNA by 80%. As expected, CLI-095 also inhibited TLR4-dependent LPS induction of IFNβ (Figure 3b). These data further support the finding that HA fragments induce IFNβ via a TLR4-dependent pathway.

### HA fragments induce IFNβ independent of MyD88 via TRIF

**Figure 4:**
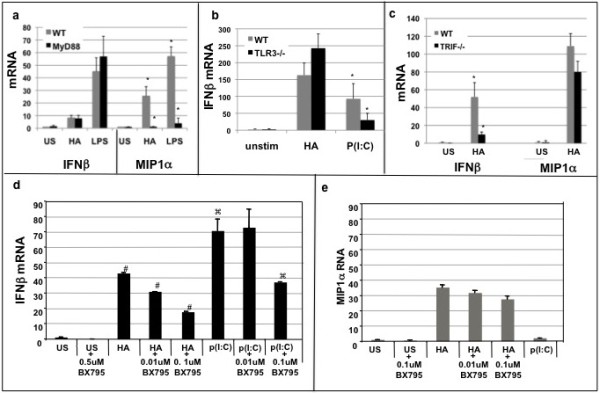
**HA fragment induction of IFNβ is independent of MyD88, and TLR3 but dependent upon TRIF and TBK1.** Thioglycollate elicited peritoneal macrophages from WT, MyD88 null, TLR3, TRIF null mice were stimulated with 200 ug/mL LMW HA fragments, LPS 10 ng/ml or p(I:C) 5 ug/ml for 3 hours; cells were collected in Trizol, RNA extracted, and cDNA was analyzed with real-time PCR (**a**) HA fragments require MyD88 to induce MIP1α but NOT IFNβ. (**b**) TLR3 is not necessary for HA induced IFNβ mRNA. (**c**) In the absence of TRIF, HA fragments induced significantly less IFNβ. (**d**,**e**) RAW macrophages were stimulated with 200 ug/mL HA fragments for 3 hours +/- the TBK1 inhibitor BX795; RNA was isolated and Real-time PCR performed. HA fragments and p(I:C) induced significantly less IFNβ in the presence of the TBK1 inhibitor but HA induced MIP1α was not significantly inhibited. Data reported as fold induction over WT unstimulated; data are mean of 3-7 experiments run in triplicate; * p < 0.05 HA vs US; # p < 0.05 HA vs HA + BX795; p < 0.05 p(I:C) vs p(I:C) + BX795.

The ability of HA fragments to induce IFNβ in a TLR4 dependent fashion was somewhat surprising. Previously, it has been shown that LMW HA fragments induced inflammatory gene expression via MyD88 [8,9,18]. However, IFNβ expression via TLR3 and TLR4, proceeds via MyD88-independent pathways [19,21,22]. Thus, we sought to determine whether HA fragment induction of IFNβ is MyD88 dependent. Thiolglycollate-elicited primary macrophages were harvested from MyD88 null and wild type mice, stimulated in serum-free media with 200 ug/mL of HA fragments, RNA was extracted and analyzed with real time PCR. HA fragments induced similar levels of IFNβ mRNA in both MyD88 null and WT macrophages (Figure 4a). In contrast, the production of the inflammatory cytokine MIP1α was reduced in the MyD88 null mice (Figure 4a). These data demonstrate that HA-induced IFNβ proceeds via a MyD88-independent pathway, and thus identifies a novel signaling pathway responsible for HA fragment-induced inflammatory gene expression.

As IFNβ is known to be induced by TLR3, we wanted to insure that HA induction of IFNβ via TLR4 was not also utilizing TLR3 [3]. Therefore, thioglycollate-elicited primary macrophages were harvested from TLR3 null and WT mice, stimulated in serum free media with 200 ug/mL of HA fragments for 3 hours, and RNA was extracted and analyzed with real time PCR. HA fragments equally induced IFNβ mRNA in both WT and TLR3 null macrophages (Figure 4b). Thus, HA fragment-induced IFNβ is not dependent on TLR3.

Since HA did not require MyD88 for induction of IFNβ via TLR4, we hypothesized that HA fragment-induced IFNβ expression required the TIR-domain-containing adapter-inducing interferon-β adaptor protein (TRIF). Thiolglycollate-elicited primary macrophages were harvested from TRIF null and WT mice, stimulated in serum free media with 200 ug/mL of HA for 3 hours, RNA was extracted and analyzed with real time PCR. HA fragment induction of IFNβ mRNA was markedly reduced in TRIF null macrophages (Figure 4c). In contrast, the production of inflammatory cytokine MIP1α by HA fragments was not affected in the TRIF null macrophages. Thus, LMW HA induces IFNβ via a TLR4-TRIF-dependent pathway.

IFNβ is also known to be induced via a TLR-TBK1 pathway [3]. As the TLR3 receptor was not required in our model, we wanted to determine if TBK1 was part of the HA fragment-induced TLR4-TRIF induction of IFNβ. Macrophages were stimulated with 200 ug/mL HA for 3 hours with and without the TBK1 inhibitor BX795; RNA was isolated and real time PCR performed. HA fragments and poly(I:C) induced significantly less IFNβ in the presence of the TBK1 inhibitor (Figure 4d and e) but HA-induced MIP1α was not significantly inhibited. Thus, HA induced IFNβ via a TLR4-TRIF-TBK1 dependent pathway.

### HA fragments induce phosphorylation of IRF-3

**Figure 5:**
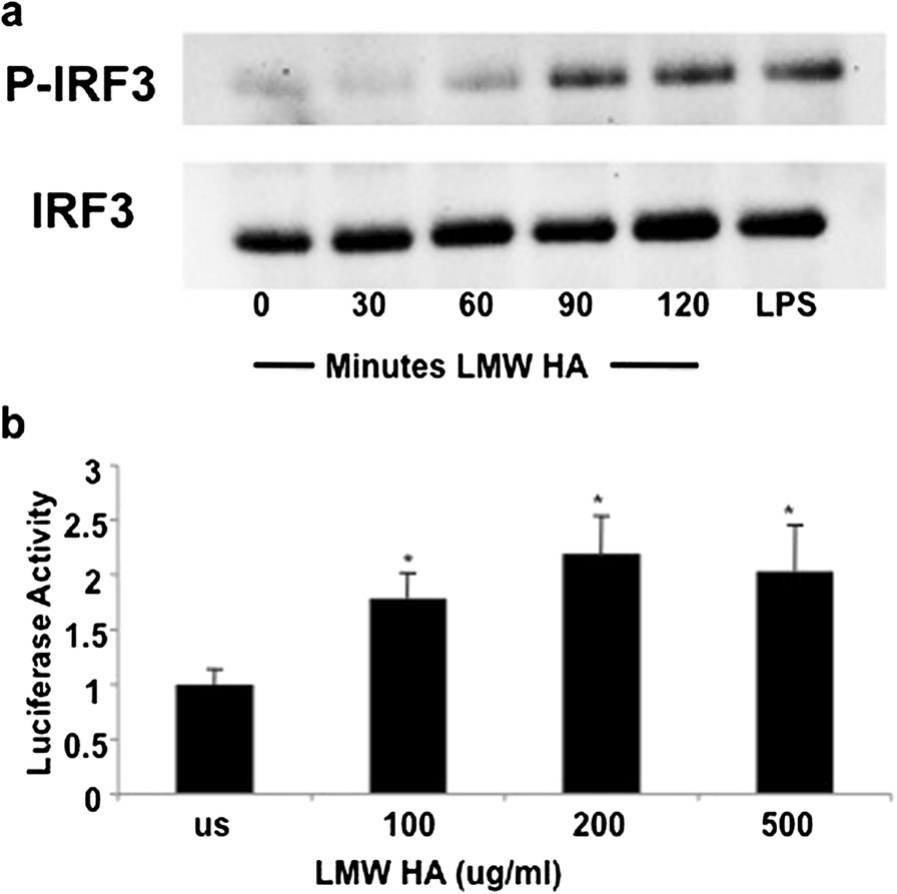
**HA fragments activate IRF-3 phosphorylation and IFNβ gene activity.** (**a**) MH-S macrophages were stimulated with LMW HA fragments, nuclear extracts were collected and analyzed for phosphorylated IRF-3 via Western blot. (**b**) RAW 264.7 macrophages were transfected with human *IFNβ* promoter driven luciferase construct and stimulated with HA fragments for 18 hours prior to luciferase measurement. HA fragments induce the IFNβ promoter by over 2 fold. These data are representative of three identical experiments. * p < 0.02 vs unstimulated.

Interferon response Factor 3 (IRF-3) is central to the production of interferon beta via the TLR3 and TLR4 pathway, while IRF7 appears to be dominant in the TLR9 signaling that predominates in plasmacytoid dendritic cell [27]. To determine the role of IRF-3 in HA fragment induction of IFNβ, we performed western analysis of HA-stimulated macrophages and interrogated extracts for members of the IRF family. MH-S alveolar macrophages were stimulated with LMW HA (200 ug/mL) for 0, 30, 60, 90, and 120 minutes; nuclear extracts were prepared, and analyzed by western blot with actin as a loading control. Only IRF-3 was phosphorylated after HA fragment stimulation, with peak phosphorylation after 90 minutes (Figure 5a).

To demonstrate the functional consequences of IRF-3 phosphorylation we evaluated the ability of HA to stimulate an IRF-3-dependent IFNβ-promoter-driven luciferase reporter construct. Cells were transiently transfected with the reporter construct and stimulated with HA fragments for 18 hours prior to cell extract isolation and IFNβ gene activity was determined by luciferase production. Transfected cells stimulated with LMW HA showed a dose-dependent increase in activation of the IFNβ gene (Figure 5b). These functional data support our model that HA fragments stimulate IFNβ expression in part through the activation of IRF-3.

## Discussion

Hyaluronan (HA) is a glycosaminoglycan that plays an essential role in tissue integrity and water homeostasis [7]. During inflammation or tissue injury, the normally high molecular weight HA is broken down into low molecular weight fragments that induce inflammatory gene expression in macrophages, dendritic cells, T cells and epithelial cells [13-15,28]. HA fragments rapidly activate the innate immune response upon tissue damage even in the absence of or prior to the establishment of infection. Thus, we have proposed the HA fragments act as endogenous danger signal [9]. We now demonstrate that as an early danger signal HA fragments also induce Type I interferons, which play a critical role in establishing anti-viral immune responses.

**Figure 6:**
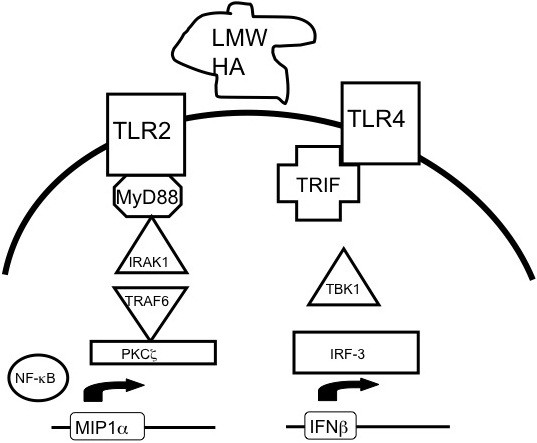
**HA fragment induced inflammatory gene via distinct pathways.** Schema of the pathways by which LMW HA fragments induces inflammatory genes via TLR2-MyD88-IRAK1-TRAF6-PKCζ-NK-κB or via TLR4-TRIF-TBK1-IRF3.

Furthermore, our studies identify an additional signaling pathway by which HA induces inflammatory gene expression. While it had previously been shown that HA fragments induced inflammatory gene expression is dependent upon MyD88 signaling, we now demonstrate a novel MyD88-independent TLR4-TRIF-TBK1 pathway for HA fragments induced IFNβ expression [9] (Figure 6). Thus our studies not only expand our understanding of the breadth of the inflammatory program induced by HA but also the intracellular signaling pathways employed by this endogenous inflammatory mediator. Our data further highlight the ability of this matrix component to modify the inflammatory milieu simultaneously via several distinct pathways.

Hyaluronan is produced by three isoforms of hyaluronan synthases and released from the plasma membrane into the extracellular space predominantly by fibroblasts [29]. It abounds in synovial and vitreous fluids, and makes up 80% of the glycosaminoglycan in the lung [7,10,30]. In a healthy lung, HA exists predominantly in a high molecular weight form that is immunosuppressive by a variety of mechanisms; it enhances suppressive T regulatory cells, inhibits macrophage phagocytosis, and is important in maintaining distribution of plasma proteins [7,10,30,31]. However, low molecular weight fragments of HA produced both by breakdown of high molecular weight forms and by direct synthesis, have profound biological effects that oppose these pro-homeostatic effects [8,9,11]. The accumulation of HA fragments is itself a nonspecific response to lung injury.

Increased levels of HA fragments in the lung, at levels similar to the doses used in these experiments, are associated with a diverse set of injuries including ventilator-induced lung injury, and bleomcyin and ozone exposure [32-36]. Mice lacking CD44, a major receptor for HA, have impaired clearance of HA fragments, and increased bleomcyin injury [11]. HA fragments instilled into the lung increases airway hyper-responsiveness in a CD44-dependent manner [33]. HA fragments have been shown to mediate airway hyper-responsiveness seen with ozone exposure through both CD44 and TLR4 [33]. HA fragments promote production of inflammatory chemokines: MIP1α, MIP1β, KC, RANTES, MCP-1 and IP-10, as well as cytokines such as IL-8, IL-12 and TNF, via TLR2-MyD88-IRAK–PKζ binding [12-15]. In the bleomycin model of lung injury, mice lacking TLR2 are protected, while TLR2/TLR4 double null mice have increased mortality, suggesting that HA fragment-TLR2/TLR4 interactions have complex downstream effects [8,37,38]. The complete actions of HA fragments in the lung and other sites of tissue injury are still incompletely understood.

The generation of type I interferons is important not only locally but also systemically to condition and recruit immune cells to the site of infection [39]. The recognition of viral components and release of interferons is known to be mediated by TLR3, TLR7,TLR8 and TLR9 signaling as well as by more recently defined receptors such as RIG-I, MDA5 and DAI [39]. In addition, TLR4 signaling (for example by LPS) can lead to interferon production in a pathway dependent on STAT1 and the transcription factor IRF-3 [3]. TLR4 also uses an additional adaptor protein, TRAM, which seems to be unique to TLR4-induced interferon production [3,19,27,40]. In contrast, TLR2 ligands in general do not appear to phosphorylate STAT1 or lead to significant production of interferon beta in the majority of cases [3,19,23,27,40]. Here we demonstrate that LMW HA can increase production of IFNβ. This effect is not via the ability of LMW HA to activate TLR2 (as has been previously described) but rather by TLR4 activation. Furthermore, unlike other LMW HA signaling, LMW HA-induced TLR4 activation depends on TRIF and not MyD88.

In a study on the effects of HA on neutrophils, Leu et al. observed that apoptosis of neutrophils is decreased in TLR4 null mice [41]. They demonstrated that was due to IFNβ mediated TRAIL-TRAILR interactions, as the addition of IFNβ led to an increase in TRAIL/TRAILR in inflammatory neutrophils. Administration of intratracheal HA fragments (2000 ug/ml) was found to result in an increase in IFNβ in whole lung neutrophils, an effect that was mitigated in the TLR4-/- mice. Our studies now provide a biochemical mechanism for these observations. Our data demonstrate that LMW HA directly induces IFNβ production and that this occurs via a newly defined TLR4-TRIF-TBK1-IRF3 pathway. Thus, tissue damage causing fragmentation of matrix hyaluronan generating local high concentrations of HA fragment-induced IFNβmay prime the innate response for a potential viral infection (Figure 6), expanding the range of the ‘danger signal’ properties of LMW HA. The induction of IFNβ by HA, an endogenous danger signal, raises the possibility that the anti-viral effects of the interferons may be triggered early in injury, perhaps priming the immune system to launch a full anti-viral program.

Alternatively, the production of IFNβ may be intended to modify the pro-inflammatory effects of HA. Although the type I interferons are best known for their effects in viral infection, they also have the ability to inhibit inflammatory responses [6]. For example, in the eye, IFNβ appears to be important in suppressing inflammatory responses; IFNβ is produced in substantial amounts by retinal pigmented epithelial cells, and eliminates production of T cell chemoattractant CXCL9 in response to TNFα/ IFNg/IL-1β [42]. Moreover, in a murine model, the increase in type I interferon ten days after influenza infection led to significantly decreased neutrophilic responses to subsequent bacterial pneumonia and increased mortality [43]. Therefore, HA induced activation of IFNβ via TLR4-TRIF-TBK1 may also act as a potential brake for innate inflammatory responses. Taken together, a model emerges whereby immediate tissue damage can lead to HA fragment-induced IFNβ that primes the innate response for a potential viral infection (Figure 6). Alternatively, persistent tissue damage leading to the accumulation of HA fragments may in fact serve to down regulate certain inflammatory responses. Thus this novel downstream effect of HA expands the role of this endogenous danger signal, and opens avenues for further investigation. In addition, the redundancy of inflammatory pathways triggered by LMW HA may also add to the robustness of an inflammatory response.

## Conclusions

Low molecular weight fragments of the extracellular matrix component hyaluronan use many pathways to activate the immune system and modulate inflammation. We demonstrate a novel CD44 and MyD88 independent pathway for HA fragments to activate macrophage production of interferon-β via TLR4-TRIF-TBK1-IRF3. Our data implicate HA fragment-induced interferons resulting from tissue damage as a novel potential endogenously derived warning system initiating an anti-viral response prior to the establishment of infection. Furthermore, this HA-induced gene expression is the result of a newly defined HA fragment downstream signaling pathway.

## Abbreviations

HA: Hyaluronan; IFNβ: Interferonβ; LMW: Low molecular weight; HMW: High molecular weight.

## Competing interests

The authors declare that they have no competing interests.

## Authors’ contributions

KEB planned, performed, and analyzed most of the experiments and prepared the manuscript SLC, RSH, MJH, YC-L, and RWH assisted in planning and performing many of the experiments, JDP assisted in planning experiments and manuscript preparation, MRH planned experiments, analyzed data and prepared the manuscript. All authors read and approved the final manuscript.
